# Microscale drivers of summer CO_2_ fluxes in the Svalbard High Arctic tundra

**DOI:** 10.1038/s41598-021-04728-0

**Published:** 2022-01-14

**Authors:** Marta Magnani, Ilaria Baneschi, Mariasilvia Giamberini, Brunella Raco, Antonello Provenzale

**Affiliations:** 1 Institute of Geosciences and Earth Resources, Via Valperga Caluso 35, 10125 Turin, Italy; 2 University of Turin and INFN, Via Pietro Giuria 1, 10125 Turin, Italy; 3 Institute of Geosciences and Earth Resources, Via Moruzzi 1, 56124 Pisa, Italy

**Keywords:** Carbon cycle, Carbon cycle, Carbon cycle, Climate and Earth system modelling

## Abstract

High-Arctic ecosystems are strongly affected by climate change, and it is still unclear whether they will become a carbon source or sink in the next few decades. In turn, such knowledge gaps on the drivers and the processes controlling CO_2_ fluxes and storage make future projections of the Arctic carbon budget a challenging goal. During summer 2019, we extensively measured CO_2_ fluxes at the soil–vegetation–atmosphere interface, together with basic meteoclimatic variables and ecological characteristics in the Bayelva river basin near Ny Ålesund, Spitzbergen, Svalbard (NO). By means of multi-regression models, we identified the main small-scale drivers of CO_2_ emission (Ecosystem Respiration, ER), and uptake (Gross Primary Production, GPP) in this tundra biome, showing that (i) at point scale, the temporal variability of fluxes is controlled by the classical drivers, i.e. air temperature and solar irradiance respectively for ER and GPP, (ii) at site scale, the heterogeneity of fractional vegetation cover, soil moisture and vegetation type acted as additional source of variability for both CO_2_ emissions and uptake. The assessment of the relative importance of such drivers in the multi-regression model contributes to a better understanding of the terrestrial carbon dioxide exchanges and of Critical Zone processes in the Arctic tundra.

## Introduction

Accelerated warming in the Arctic is a reason for concern^1^ especially because a large amount of carbon from permafrost can become available in deglaciated soils^2–4^. Temperature rise and permafrost thaw are expected to accelerate soil organic carbon decomposition and therefore its respiration to CO_2_^5–9^ and/or its export to the Arctic Ocean^9–11^. On the other hand, the increase of snow-free days and higher temperatures can favour vegetation growth and the associated carbon uptake^12,13^.

The effects of these opposing processes make the future Arctic carbon budget difficult to assess. Further uncertainties come from a possible shift of the vegetation community towards shrubs or late successional species^14,15^, biomass loss due to vegetation stress and disturbance^16,17^, increase of cloud cover in summer^18^ and persistent nutrient limitation, especially in the High Arctic^19,20^.

Current field data do not solve the issue: annual measurements around the Arctic suggest that the tundra behaves either as a weak carbon sink^21,22^, as a source^23^, that the annual balance is close to zero^24^, or that high interannual variability masks the true long-term behavior^25^. Such large uncertainties on the estimate of carbon fluxes hamper a reliable prediction of the expected effects of climate change^26^, and a better understanding of the Arctic carbon drivers is urgently needed. To contribute to this endeavour, here we focus on measurement of CO_2_ fixation by photosynthesis and emission by vegetation and soil respiration, and implement a data-driven model able to identify the main flux drivers.

Empirical approaches have the advantage of exploiting the direct link with data. In this biome, however, difficulties in modelling the soil–vegetation–atmosphere carbon exchanges often arise from the heterogeneity of the tundra vegetation cover. At landscape scale, environmental conditions can vary within a few meters, creating a spatially diversified ensemble of vegetation patterns^27^, composed of different plant communities that may in turn influence the local conditions, such as soil moisture^28^. In such a patchy ecosystem, landscape-scale (or finer-scale) studies allow to identify the relevant carbon flux drivers, isolating individual ecosystem components and assessing their interactions.

**Figure 1:**
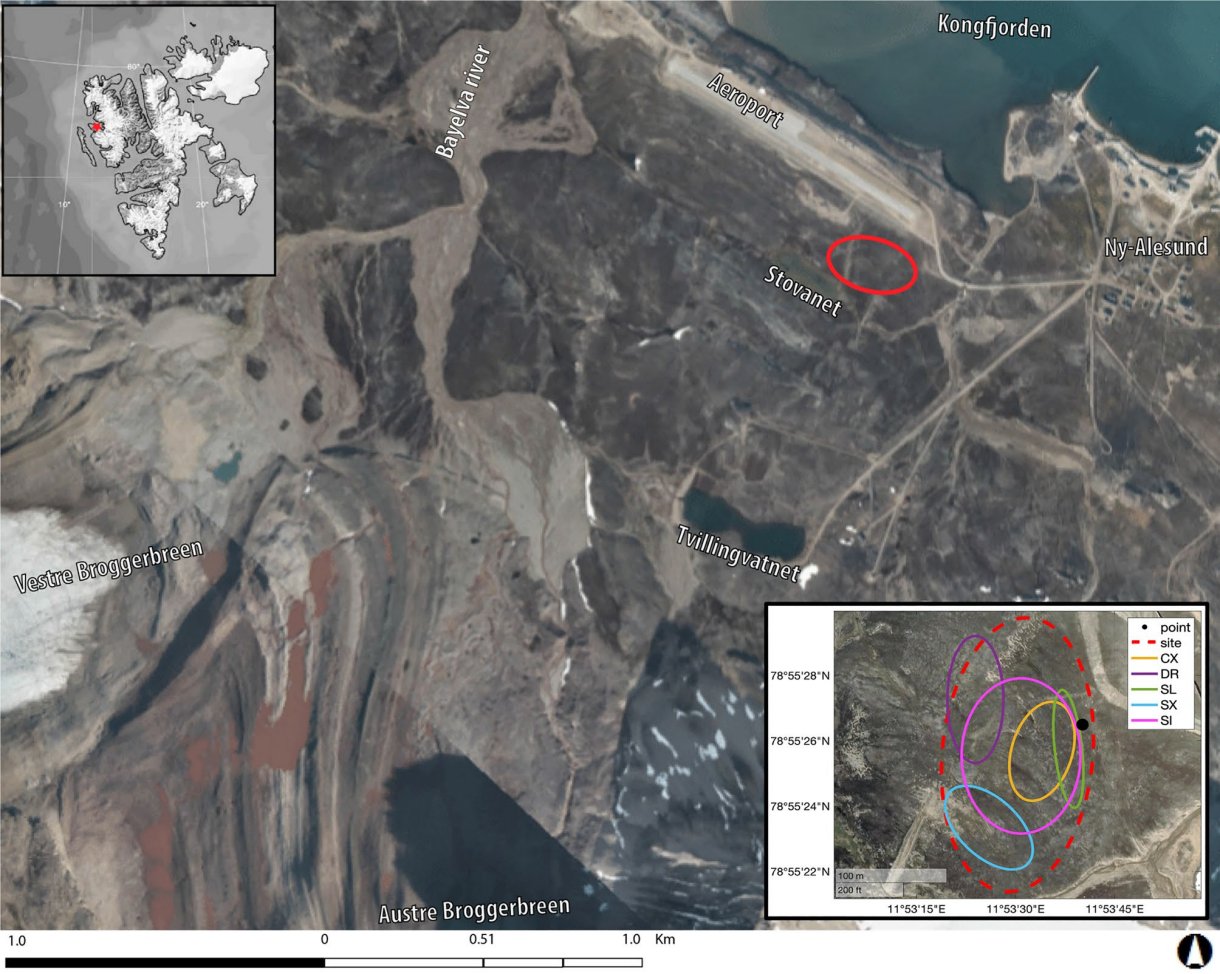
Location of the study site (red circle) with indication of toponyms. Aerial/satellite image modified from TopoSvalbard (courtesy of Norwegian Polar Institute, https://toposvalbard.npolar.no/). Top-left inset: location of study site (red star) in Spitsbergen, Svalbard Islands (NO). Bottom-right inset: distribution of the sampling areas for point-scale (black dot), site-scale (red dashed line) and species-specific (solid lines) samplings. Satellite basemap available in MATLABR2020a, hosted by Esri.

Although it is known that CO_2_ emissions depend on air or soil temperature^29^ and that uptake by photosynthesis depends on solar radiation^30^, the whole complex of environmental and physiological drivers influencing fluxes at fine scale is not fully understood. In addition, dissimilar functional dependences on the drivers were obtained in the different studies, and a quantification of the driver relevance across different scales was often missing, further enhancing the uncertainties in the assessment of the tundra carbon budget^31^. Here, we address such gaps, by exploring what are the drivers of the short-term temporal variation of CO_2_ fluxes at a fixed point, what factors contribute to the spatial–temporal variability of CO_2_ fluxes at site scale, and what are the differences—if any—between different vascular plant species. To achieve these goals, we performed in situ samplings of CO_2_ fluxes, basic meteoclimatic variables and vegetation characteristics in the High-Arctic tundra of the Bayelva basin, Svalbard Islands (NO), during summer 2019, in the framework of our general study of the Arctic Critical Zone^32^. Figure 1 shows a map of the Bayelva study site.

Carbon dioxide fluxes were measured by means of a portable flux chamber that allowed us to analyze the effects of vegetation patchiness and of local environmental variations. In addition, the estimate of the green fractional cover from digital RGB images and vegetation identification were used to assess the contribution of plants to the carbon dioxide exchanges. We designed three different sampling sets, aimed at investigating: (1) the temporal variability of CO_2_ fluxes, by performing 24-h measurements at a fixed sampling point (*point-scale samplings*); (2) the spatial–temporal CO_2_ variability at the site scale (*site-scale samplings*), sampling in points randomly distributed over the sampling site, which covered an area of about 22,000 m^2^; (3) whether and how different vegetation types affect the fluxes (*species-specific samplings*), by performing measurements in points covered with the 5 most representative vascular species in the catchment. We identified the drivers of summer CO_2_ emission and uptake by means of statistical analysis, building regression models that reproduced CO_2_ fluxes with large explained variance. In addition, we assessed the weight of each driver included in the regressions, identifying the most relevant variables. The results of our modelling effort indicated what are the mandatory drivers to be monitored in the field, and can be used to inform the implementation of specific process-based models addressing the relevant variables.

## Results

### Point-scale samplings

**Figure 2:**
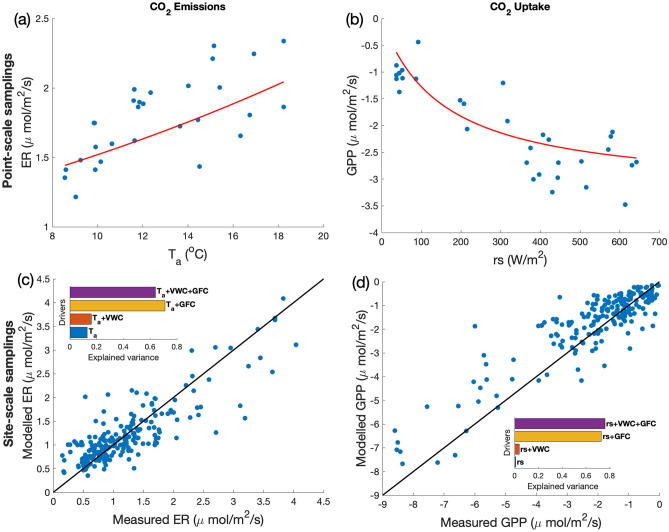
Upper panels: Plots of (**a**) ER versus T_a_ and (**b**) GPP vs rs for point-scale samplings, with red lines corresponding to best-fit curves, according to Eqs. (1) and (2). Lower panels: Plots of the measured versus modelled ER (**c**) and GPP (**d**) for site-scale samplings. Modelled values as obtained from Eqs. (3, 4). Parameters and statistics as in Table 1. Inset: explained variance for models including only the listed drivers.

*Point-scale samplings* were performed for 24 h across 2 days of clear sky and stable meteorological conditions at a fixed point, mostly covered with *Carex* spp. The CO_2_ fluxes and the meteorological variables displayed a hump-shaped temporal behavior during the measurement days (see Fig. 2; S2 in the Supplementary Material), with the only exceptions of air pressure, that showed a slightly decreasing trend with average value of 1023 hPa and fluctuations comparable with measurement errors, and soil moisture, that varied irregularly preventing the identification of a clear temporal evolution. The pattern of Ecosystem Respiration (ER) and Gross Primary Production (GPP) matched those of their classical drivers, i.e. air temperature (${T}_{a}$) and solar irradiance (*rs*). A net uptake (i.e. negative Net Ecosystem Exchange, NEE) was observed during the day, between about 3:00 and 18:00 UTC, and net emission (i.e. positive NEE) in the remaining hours, when solar irradiance dropped below 100 W/m^2^. Total night darkness is absent during Arctic summer and therefore null irradiance was never attained, with a minimum measured irradiance of 37.12 W/m^2^ over the whole campaign.

The classical dependence of carbon emission on temperature is modelled as an exponential^29^, while the dependence of carbon uptake by photosynthesis is modelled as a double-hyperbolic function of solar radiation^30^. These are expressed as:

1 $$ER = a \exp \left( {b T_{a} } \right) + \varepsilon ,$$

2 $$GPP = \frac{{F_{\max }\, \alpha rs}}{{F_{\max } + \alpha rs}} + \varepsilon$$

where *a* is a free parameter, corresponding to ecosystem respiration at 0 °C, *b* is related to the usual *Q*_*10*_ factor^29^ as *Q*_10_ = exp (10* b*), *F*_*max*_ is the maximum photosynthetic flux for infinite light supply and α is the apparent quantum yield. These regression parameters are to be fitted from data, while $\varepsilon$ represents the residual of the fit, that includes all the unmodelled dependences and the stochastic contribution of Gaussian noise.

At point-scale, the temporal variations of ER and GPP were reproduced by the classical dependences (1) and (2), with large values of the explained variance (0.73 for ER and 0.86 for GPP), as shown in Fig. 2a, b. Best-fit parameter values were $a = 1.06\,\upmu {\text{mol/m}}^{2} {\text{/s}}$ and $b = 0.036\,^{ \circ } {\text{C}}^{ - 1}$ in Eq. (1), and $F_{\max } = - 3.23\, \upmu {\text{mol/m}}^{{2}} {\text{/s}}$ and $\alpha = - 0.021\, \upmu {\text{mol/W/s }}$ in Eq. (2)*.* For both emission and uptake, the introduction of additional variables did not improve the performances of the models in terms of AIC (Akaike Information Criterion^33,34^, see the section “Regression Models” in “Methods”), which was − 13.21 for ER and − 35.25 for GPP. Residuals were gaussian (Lilliefors’ test^35^), and heteroscedastic (Bartlett’s test^36^). Therefore, the hourly temporal evolution of CO_2_ fluxes at point-scale was best explained by the temporal variation of air temperature and solar irradiance, respectively.

### Site-scale samplings

For *site-scale samplings*, we performed a total of 177 individual (point-scale) measurements during the 10 days of the campaign. For this ensemble of measurements, the classical models (1) and (2) showed very low explained variances (${\sigma }_{expl}^{2}$) for both ER and GPP (see the insets of Fig. 2c, d). In the multi-regression models (M1) and (M2), described in the Methods section, Green Fractional Cover (GFC, see Fig. S1 in the Supplementary Material) and Volumetric Water Content (VWC) were selected as additional predictors for the fluxes. The models identified in this way are:

3 $$ER = \left( {a_{0} + a_{1} GFC + a_{2} VWC} \right)\exp \left( {b_{0} T_{a} } \right) + \varepsilon ,$$

4 $$GPP = \frac{{F \, \alpha_{0} \, rs}}{{F + \alpha_{0}\, rs}} \left( {A_{0} + A_{1} GFC + A_{2} VWC} \right) + \varepsilon ,$$

and the corresponding parameter values are reported in Table 1. Here, $\varepsilon$ represents the (random) residuals of the model. The temperature response parameter in the ER model, ${b}_{0}$, corresponds to ${Q}_{10}=$ 1.6, well within the range of published estimates for the Arctic tundra^37–40^.

**Table 1:** Parameters of the best-fit multi-regression models of Eqs. (3) and (4) and related statistics, as obtained for the ensemble of site-scale samplings (‘All’), vascular vegetation (‘V’), non-vascular vegetation (‘NV’) and the mix of vascular and non-vascular vegetation (‘MIX’).

Parameter	Units	Vegetation class			
		All	V	NV	MIX
*a*_0_	μmol/m^2^/s	0.17	0.25	0.35	0.33
*a*_1_	μmol/m^2^/s	2.63	2.27	3.24	1.12
*a*_2_	μmol/m^2^/s	0.0035	0.008	0.007	0.005
*b*_0_	℃^−1^	0.074	0.039	0.19	0.042
*A*_0_		0.021	0.13	0.29	0.38
*A*_1_		7.31	3.88	17.89	4.18
*A*_2_		0.0024	0.0099	0.013	0.0027
*F*	μmol/m^2^/s	−2.16	−4.39	−0.78	−2.83
${\alpha }_{0}$	μmol/W/s	−0.031	−0.042	−0.0041	−0.015
$\sigma$_*exp*_^2^ Eq. (3)		0.64	0.69	0.19	0.26
*AIC* Eq. (3)		−159.51	−25.26	2.76	−25.98
$\sigma$_*exp*_^2^ Eq. (4)		0.76	0.73	0.33	0.39
*AIC* Eq. (4)		−217.76	−26.00	−2.98	−43.66

Figure 2c, d shows the modelled versus measured values for the best-fit model, respectively for ER and GPP, and the variance explained by different combinations of the flux drivers. The GFC caused the largest enhancement of ${\sigma }_{expl}^{2}$ compared to the classical models (${\sigma }_{expl}^{2}$ increased of + 0.58 and + 0.72 respectively for ER and GPP), while the model enhancement due to VWC was smaller (${\sigma }_{expl}^{2}$ increased by + 0.03 in both ER and GPP models). Residuals were gaussian, according to Lilliefors’ test^35^, and heteroscedastic, according to Bartlett’s test^36^.

**Figure 3:**
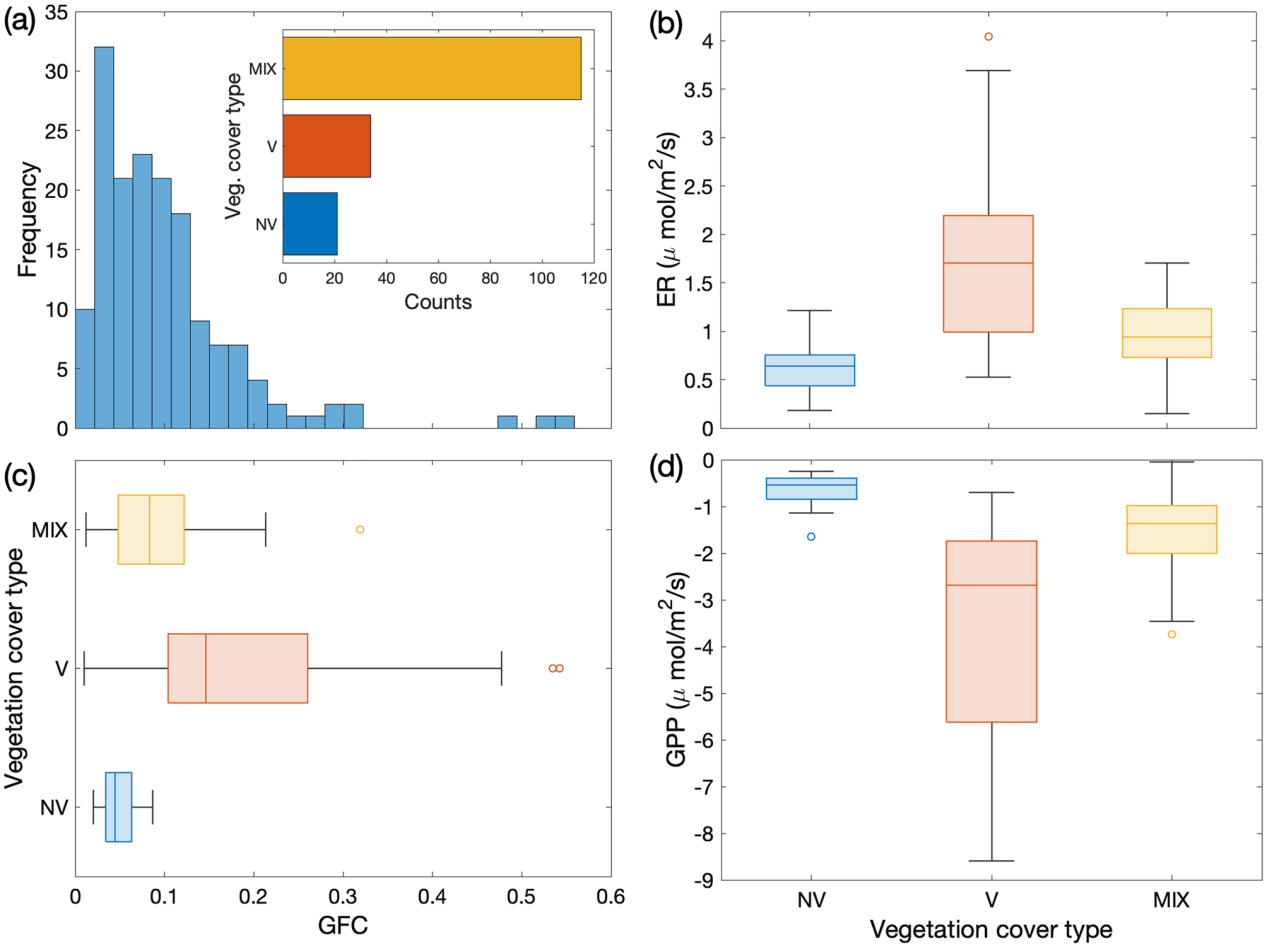
(**a**) Frequency of the Green Fractional Cover, GFC, in site-scale samplings including all types of vegetation cover. Inset: Number of points of the different vegetation types in site-scale samplings: (V) vascular species; (NV) non-vascular species; (MIX) mix of vascular and non-vascular species. Box plots of (**b**) Ecosystem Respiration, ER, (**c**) GFC and (**d**) Gross Primary Production, GPP, for V, NV and MIX vegetation classes. Box plots display the median (line), the lower and upper quartiles (box), outliers computed using the interquartile range (points) and the minimum and maximum values that are not outliers (bars).

As shown in Fig. 3a, the distribution of the GFC was dominated by small values, mostly confined below 0.20, with sporadic samplings characterized by a larger green vegetation cover. Large values of GFC were mostly associated with sampling points where the cover was dominated by vascular species, class V, while moss, lichens and bacterial soil crust, included in class NV, showed the lowest GFC values (Fig. 3c). Only 4 points (i.e., 2% of the total sample size) were classified as bare soil, class BS, and therefore were not included in the following analysis, being a minor component in this environment (see CAVM-classification in the Method section). Significant differences were found between the mean GFC of V and NV classes (*p* = 0.001) and larger variability was associated with V compared to NV (boxes and bars in Fig. 3c). Sampling points where a clear predominance of V or NV species was not present (class MIX) showed a significantly different mean value of GFC compared to both V (*p* = 0.03) and NV (*p* = 0.02) classes, with mean value and variability in between the V and NV ones. Significant differences between vascular, non-vascular and mixed classes were obtained also in the ER and GPP mean values (Fig. 3b–d), where again we observed larger variability and stronger average fluxes associated with vascular species, compared to non-vascular and mixed ones. Average ER for NV and MIX classes were consistent with measurements performed by Chae et al. at the same site^41^, and the variability range of both fluxes of NV (bars in Fig. 3b–d) agrees with that observed at the Eddy Covariance site of Lloyd^42^, which is dominated by non-vascular vegetation.

**Figure 4:**
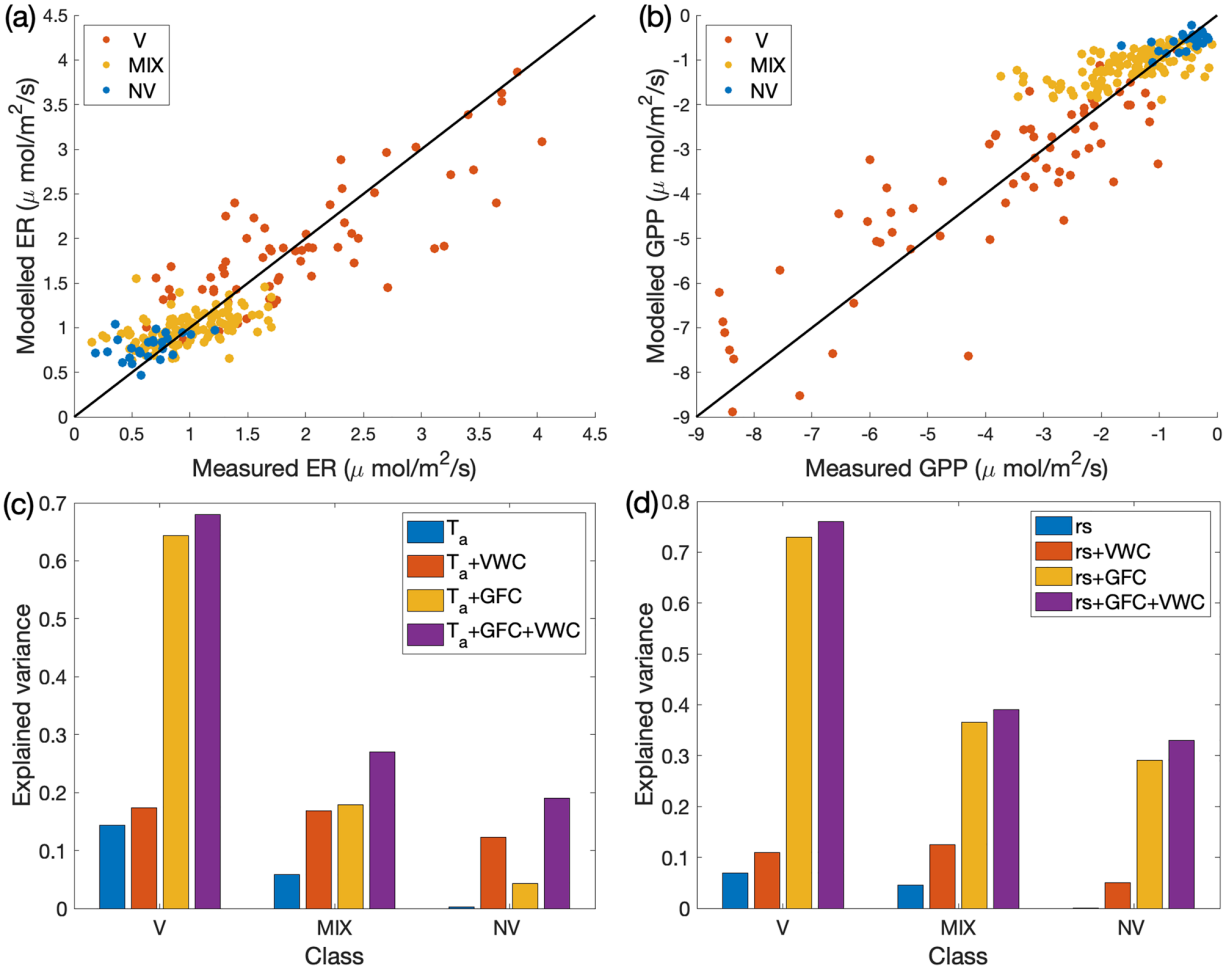
Upper panels: Plots of the measured versus modelled ER (**a**) and GPP (**b**) for vascular (V), non-vascular (NV) and mixed (MIX) vegetation classes. Lower panels: (**c**) ER explained variance for model (3), and (**d**) GPP explained variance for model (4), including only the listed drivers for the three classes.

Empirical models separately derived for the V, NV and MIX cases led to the identification of the same driving variables indicated in Eqs. (3, 4). As expected, the maximum photosynthetic rate, *F*, was higher for V compared to NV and MIX^42^. However, the explained variance drastically lowered passing from V to MIX to NV for both ER and GPP (Table 2; Fig. 4a–d). Vascular plants were characterized by explained variances that were similar to those identified for the whole set of measurements (‘All’ in Table 2). In contrast, non-vascular and mixed vegetation showed explained variances lower than 0.50 and none of the other measured variables enhanced the model representativeness. The presence of GFC as one of the drivers for NV fluxes is presumably due to the presence of (green) mosses, and to the fact that assigning a point measurement to one of the vegetation classes was based on the prevailing cover type, which cannot exclude the occurrence of small subdominant individuals of vascular species in points classified as NV. In any case, the increase in explained variance generated by the introduction of GFC in the multi-regression model decreased from V to MIX to NV (Fig. 4c, d). Conversely, the contribution of VWC increased from V to MIX to NV (Fig. 4c, d).

**Table 2:** Parameters of the multi-regression models and related statistics for the species: *Carex* spp. (CX), *Dryas octopetala* (DR), *Salix polaris* (SL), *Saxifraga oppositifolia* (SX), *Silene acaulis* (SI) and all the 5 species merged together (all spp).

Parameter	Units	Vegetation species					
		CX	DR	SL	SX	SI	All spp
*a*_0_	μmol/m^2^/s	0.92	0.46	0.18	0.11	0.22	0.18
*a*_1_	μmol/m^2^/s	0.48	0.69	1.48	0.97	0.57	1.61
*a*_2_	μmol/m^2^/s	0.0032	0.0014	0.005	0.0032	0.0020	0.0034
*b*_0_	℃^−1^	0.044	0.082	0.054	0.097	0.132	0.071
*A*_0_		0.41	0.13	0.34	0.20	0.19	0.018
*A*_1_		2.28	2.47	3.77	5.77	2.18	3.63
*A*_2_		0.0084	0.0014	0.0052	0.0019	0.0003	0.0042
*F*	μmol/m^2^/s	−7.90	−6.15	−5.38	−2.95	−20.63	−6.06
${\alpha }_{0}$	μmol/W/s	−0.011	−0.059	−0.014	−0.017	−0.022	−0.024
$\sigma$_*exp*_^2^ Eq. (3)		0.63	0.66	0.65	0.63	0.74	0.70
*AIC* Eq. (3)		3.31	−9.85	−6.56	−5.56	−19.03	−109.26
$\sigma$_*exp*_^2^ Eq. (4)		0.59	0.56	0.85	0.57	0.72	0.78
*AIC* Eq. (4)		2.00	−3.54	−10.09	−1.99	−22.13	−119.67

### Species-specific samplings

Given the relevant role played by vascular vegetation in the carbon flux budget, we explored whether different vascular species were associated with a different behavior of the carbon fluxes through *species-specific samplings* focused on the local vascular species pool: *Carex* spp. (CX), *Dryas octopetala* (DR), *Salix polaris* (SL), *Saxifraga oppositifolia* (SX) and *Silene acaulis* (SI). The *species-specific samplings* were performed in the same area of the site samplings, in order to reduce the possible intraspecific and interspecific variability due to unaccountable local variations in soil chemistry that might affect the fluxes^19,20,43^. See Supplementary Material, Fig. S3 for distribution of *species-specific samplings* along the sampling campaign.

**Figure 5:**
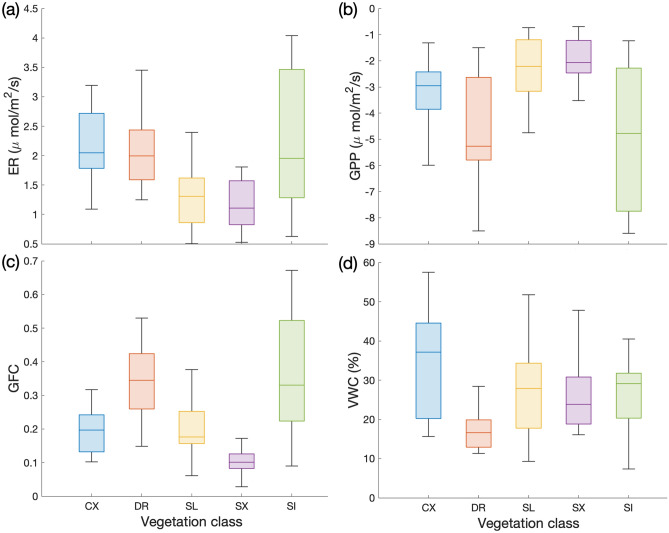
Box plot of the Ecosystem Respiration (ER, **a**), Gross Primary Production (GPP, **b**), Green Fractional Cover (GFC, **c**) and Volumetric Water Content (VWC, **d**) for species-specific samplings: CX = *Carex* spp., DR = *Dryas octopetala*, SL = *Salix polaris*, SX = *Saxifraga oppostifolia* and SI = *Silene acaulis*. Box plots display the median (line), the lower and upper quartiles (box), and the minimum and maximum values (bars).

Comparing the meteo-climatic variables and CO_2_ fluxes for each couple of species-specific measurements (see Table S1 in the Supplementary Material for differences and their significance), solar irradiance showed no significant differences between classes, with the only exception of the couple CX-SI. Significantly larger atmospheric pressure and significantly lower air humidity were recorded for CX samplings, which were indeed performed during fair, clear sky whether conditions (https://seklima.met.no/observations). DR showed significant differences in the mean soil temperature and soil moisture (also shown in Fig. 5d), compared to other species. The average green fractional cover (Fig. 5c) was significantly different between most species, except for the couples CX-SL, CX-SX, SL-SX and DR-SI. Fluxes were significantly different between most of the species, except for the couples of DR-SI and SL-SX that did not show significant differences in ER, NEE and GPP. ER was comparable (*p* > 0.05) between CX-DR, CX-SI, DR-SI and SL-SX, and NEE was comparable (*p* > 0.05) between CX-SL, CX-SX, SL-SX and DR-SI. As already observed for *site-scale samplings*, more intense (lower) GPP matched higher ER (Fig. 5a, b), and, across species, the magnitude (absolute values) of both fluxes was higher for DR and SI, although being also highly variable, while SX showed the less intense mean ER and GPP, as well as the lowest variability. Such patterns are consistent with other species-specific studies in the same catchment^44^.

Following the same procedure discussed above, we estimated a suitable empirical model for each vascular species. First, we tested the classic models (1–2), which explained flux variability to only a limited extent (${\sigma }_{expl}^{2}$ ranging from 0.32 to 0.49 for ER and from 0.08 to 0.38 for GPP), although larger explained variances were obtained in these *species-specific samplings* compared to *site-scale samplings*. Then, we turned to multi regression models, obtaining the same models as for the *site-scale samplings* (Eqs. 3, 4), where again GFC was responsible for the largest enhancement of the explained variance. Estimated parameters and statistics are reported in Table 2. Patterns of *F* among species closely follow the ones of^45^. Significant differences between some of the species were obtained in parameters related to GFC (i.e. a_1_ and A_1_, as shown in Table S2 in the Supplementary Material). In particular, the SI and DR parameters did not show any significant difference, as well as SL and SX, while CX was in between those two groups, showing significant differences in ${a}_{1}$ compared to DR, but no differences with respect to SI.

No significant correlations were found between GFC and VWC, neither for any of the species (see also Fig. S4 in the Supplementary Material), nor for the *site-scale samplings*. This excluded cross-correlations in the model, potentially resulting from the effect of the biomass on the local soil moisture (e.g., larger biomass could have higher water demand, resulting in the reduction of soil moisture in the root zone).

Merging all vascular vegetation species (column ‘All Spp’ in Table 2), the best models were again those including GFC and VWC, with explained variance comparable with that obtained for *site-scale samplings* (Table 1). Significant differences between *site-scale samplings* (‘All’ in Table 1) and the merged vegetation case (‘All Spp’ in Table 2) were observed not only in the mean value of the fluxes (*p* = 0.003 for ER and *p* = 0.005 for GPP), but also in model parameters related to GFC (*p* = 0.04 for ${a}_{1}$ and *p* = 0.01 for ${A}_{1}$), presumably owing to the contribution of non-vascular vegetation to the fluxes in the full *site-scale samplings*. This suggests that the total flux and its variability at site scale cannot be explained solely by the vascular species contribution, despite the fact that the fluxes for vascular vegetation were larger than for non-vascular vegetation. Conversely, the models estimated for the merged *species-specific samplings* and the vascular class in *site-scale samplings* did not show significant differences, confirming that the selected species properly represented the behavior of the vascular plants in this area.

## Discussion

Biological CO_2_ emissions by plant and soil respiration are known to depend on temperature^29^, while CO_2_ uptake by photosynthesis is a function of solar radiation^30^. However, other additional drivers modulate the carbon fluxes in the soil-vegetation system. Several studies proved the influence of soil moisture on CO_2_ emission and uptake in polar and Alpine tundra^46–49,49^. Soil properties, such as the active layer depth^39,46,50^, were sometimes included in the models. Biomass, green vegetated area or leaf area index (LAI) are recognized to be important drivers, describing the contribution of vegetation to the carbon fluxes^37,51^. Finally, plant functional types or species-specific regressions were used to explore the carbon fluxes resulting from diversified vegetation physiology^37,45,52^.

Here, we identified the main drivers of CO_2_ emission and uptake at the peak of the growing season, developing empirical multi-regression models able to explain a large portion of the flux variance with a minimal set of parameters. At point-scale (small circular plots with a diameter of about 0.29 m), most of the temporal variability of the fluxes at a fixed location was explained by the classical univariate dependences of ER and GPP on air temperature and solar irradiance, respectively. When zooming out to the site-scale (of the order of 150 × 150 m^2^, representative of the local vegetation community), the standard functions explained only a small fraction of the flux variability, dictating the need for multi-regression models, in which soil moisture and green fractional vegetation cover of plants emerged as essential drivers besides classical ones. In addition, the CO_2_ fluxes depended on the vegetation composition within the sampling area. This indicates that at site-scale most of the variability is related to the spatial heterogeneity of soil moisture and vegetation. It is worth noticing that the *site-scale samplings* were performed between roughly 7:00 and 19:00 UTC in different days (Fig. S3 in Supplementary material). During the site-scale sampling time the air temperature and light intensity spanned respectively between 8.2 and 17.5 °C, and 95 and 651 W/m^2^, which are comparable with the variations observed in *point-scale samplings* (Fig. 2). Hence, the relevance of the additional drivers in the site-scale study is not just due to a possibly small variability of temperature and light intensity. Conversely, these additional drivers account for surface heterogeneity and act to modify the magnitude of the fluxes’ dependence on their classical drivers.

Vegetation effects on fluxes are expected to depend on plant characteristics, such as the leaf area^51,53,54^, where plant-atmosphere gas exchange occurs via plant stomata, on the green biomass^55^, that contains chlorophyll, and on species-specific response to environmental drivers at comparable biomass^44,45^. Here, the green fractional vegetation cover (GFC) was used as a bulk descriptor of the vegetation area prone to gas exchanges. The GFC estimated from digital images provides a speditive and accurate information, useful for exploration studies. Clearly, GFC could be replaced by other on-site measurements, such as the Normalized Difference Vegetation Index (NDVI) estimated by portable NDVI sensors^37^. In general, GFC may underestimate vegetation compared to the green or leaf area index, because it does not account for wilting biomass, reddish plant elements or leaf layering. Nevertheless, this site is characterized by very limited stratification and vertical development of vegetation^45^. Only sporadic measurements were performed on wilting vegetation and solely the margin of *Saxifraga oppositifolia* showed reddish nuances, which are therefore not included in the GFC computation. On the other hand, GFC has the advantage of accounting also for moss cover, which is neglected by LAI estimates^37^.

The GFC resulted to be the strongest additional predictor of the fluxes, because it was associated with the largest enhancement in the explained variance, for both single vascular vegetation species (*species-specific samplings*) and mixed vascular and non-vascular vegetation (*site-scale samplings*). This suggests that a large portion of ER is generated by autotrophic respiration. Soil moisture (VWC) was the second significant additional predictor, combining effects from precipitation and active layer thawing (similarly to what was observed for the Alpine tundra^56^, and the Antarctic tundra^49^). Soil moisture is a recognized constraint for fluxes of poikilohydric vegetation such as mosses and lichens^39,51,57^. This was for instance suggested to be the main limit of the Eddy Covariance (EC) modelling study of Lloyd^42^ in the same site at peak season. Furthermore, soil moisture can impact carbon and nitrogen mineralization, thereby affecting CO_2_ assimilation^58^. The predictors identified here agreed with those highlighted by Li et al.^40^ and Cannone et al.^45^, for the same sampling period, including the drivers in the non-linear modelling framework. Moreover, our assessment of the drivers’ weight in the estimated models represents a unique insight in the modelling effort for the Arctic environment.

An important point concerns the role of vascular versus non-vascular vegetation. The mosaic structure of the Arctic tundra is characterized by isolated vascular species nested inside a matrix of non-vascular elements, such as lichens, mosses or bacterial soil crust^59^. Despite the significant area occupied by non-vascular vegetation, we observed that the contribution of vascular species was the most relevant to the site carbon fluxes, and vascular species showed, on average, ER and GPP that were significantly larger than the non-vascular ones. Random sampling of the surface indicated that most of our measurements involved vascular vegetation, or a surface partially covered with vascular vegetation (class ‘MIX’ in Fig. 3), suggesting that the spread of vascular plants was not negligible at this site, in keeping with Yoshitake et al.^60^. Vascular plants are late successional species in the tundra biome^57,60^ and are expected to be facilitated by the effects of climate change^14^. Higher temperatures, broadened growing period and larger water availability^1,61^ may possibly result in vascular species outcompeting pioneering vegetation^14,57^, such as lichens and mosses, and driving the carbon dioxide exchanges of the Arctic tundra in the next future.

For these reasons, we explicitly explored the differences between vascular vegetation classes that were representative of the local species pool. For each of the vegetation classes, the same drivers of *site-scale samplings* were obtained. Significant differences between model parameters related to the green fractional cover (${a}_{1}$ and ${A}_{1}$ in Eqs. 3, 4) estimated for different vegetation classes suggested a further distinction of vascular classes into functional groups, hinging in particular on the different response of carbon fluxes to the same green fractional vegetation cover. Different values of the model parameters for the same GFC indicated a species-specific response. Such results mirrored the significant differences between species, which were comparable between SI-DR and SX-SL, while CX showed hybrid characteristics between these two groups.

The observed flux differences may depend on the vegetative cycle of the species along the growing season, as for instance the flowering and seed dispersal of SL and SX occur early in the season (https://svalbardflora.no/), possibly resulting in an anticipated vegetative peak compared to other species. Our species grouping (i.e. SI-DR vs SX-SL) agrees with the results of a cluster analysis based on microhabitat for the same site^62^, possibly suggesting that plants belonging to the same group not only favour similar substrates but also display similar functioning. Previous studies also suggested that differences in fluxes can be correlated with the successional status and nitrogen content along a deglaciated transect in this catchment^44^ and with functional types related to ecosystem processes^63^. Nevertheless, all the above interpretations are based on a classification that relies on plant functioning rather than on their morphology, and a unifying modelling framework is still missing.

The main outcome of this analysis was a data-driven model of carbon dioxide emission and uptake that accounts for all the above drivers. The work presented here is one of the few attempts to build empirical models for ER and GPP variability at fine scale in the High-Arctic tundra. Interestingly, in the *site-scale samplings* we found larger explained variance for GPP compared to ER model. Similar modelling efforts focussing on the Arctic tundra also detected this gap in the ability of models to capture ER variability^37,39,46^, despite including also drivers such as thaw depth and nitrogen content. The explained variance of Eqs. (3, 4) drops for the case of non-vascular vegetation, consistently with the findings of Segal & Sullivan^39^. This may suggest that, to date, models at site to landscape scale are still limited in their ability to reproduce the processes that regulate CO_2_ fluxes from lichens and mosses. By contrast, the modelling of fluxes associated with vascular species resulted in much larger explained variance compared to non-vascular case, both at site scale and in *species-specific samplings*, with values comparable for instance with^39^. Overall, the explained variance of our model for both fluxes and in *site-scale* and *species-specific samplings* are comparable or higher than other published models for the Arctic region^37,39^, with the advantage of relying on solid theoretical assumptions on the role of additional drivers in Eqs. (3, 4). Specifically, Eqs. (3, 4) are derived from the hypothesis that additional drivers act to perturb the parameters in Eqs. (1, 2). Such hypothesis is supported by observations showing that the light-saturated photosynthetic rate, *F*_*max*_ in Eq. (2), can depend on nitrogen leaf content^44^ or LAI^53,64^, which may result from the photosynthetic capacity of plants being influenced by environmental constraints.

Clearly, the empirical models developed in this study can not be automatically applied to other Arctic sites. Here, we focused on the peak growing season ER and GPP, in the high Arctic Svalbard tundra. We remark that the tundra communities of Svalbard (P1–P2 CAVM) are also found in the northern part of Siberia and Canada^65^, a non-negligible part of the Arctic area and possibly sharing similar carbon flux dynamics. In addition, we stress that other drivers, such as soil or plant nitrogen content^39^ or thaw depth^46^, could become important over longer periods and/or in different sites. Interestingly, for a close-by site and the same vascular species Cannone et al.^45^ identified soil temperature, photosynthetic active radiation, LAI and species-specific photosynthetic capacity as the most relevant drivers during edge seasons (i.e. beginning and end of the growing season), despite their importance changing along the season and possibly between years. At a different location in Svalbard, Cannone et al.^50^ found primary drivers of ER and NEE that changed along the growing season, with LAI, surface temperature and soil moisture being the most important drivers at the season peak, in agreement with our results. Together, the similarities of our results with the findings from other sites and in different sampling periods, and the comparable explained variance with similar modelling efforts, suggest that our approach could be extended to other Arctic sites. Further studies of this type would help in representing the processes that drive the fluxes over the entire Arctic region.

Process-based models could make use of the knowledge gained from data-driven models to achieve more reliable projections. At watershed scale, data-driven models could become a useful parameterization to be incorporated in process-based models, as attempted by Uchida et al.^66^. Comparing regressive models obtained in different locations would also give information on processes and variables to be included and coupled in regional process-based models simulating the terrestrial carbon cycle^67–70^. Regional models would be suitable for long-term forecast, despite different models using different equations and different drivers for CO_2_ fluxes. In this context, we suggest that vegetation dynamics (represented for instance by the green fractional vegetation cover) and the land water cycle should be coupled with carbon dynamics in order to achieve realistic representations of the Arctic system. Projections under climate change scenarios would in particular benefit from accounting for at least two vegetation classes, namely vascular and non-vascular plants, as we showed that CO_2_ fluxes strongly depended on the relative cover of those two. In fact, quantifying the spatial distribution of vegetation types and their fractional cover, and combining this information with assessments of the vegetation respiration and photosynthetic capacity, may help in the assessment of carbon exchanges in Arctic ecosystems.

In conclusion, we recall that it is still debated whether the high Arctic tundra will behave as a carbon sink or source under changed climatic conditions. Uncertainties are not consistent between different process-based models and field data, thus leaving open issues in carbon budget assessments^71^. The origin of those uncertainties can be explored by small-scale studies that aim to unveil the processes underlying carbon fluxes in such a fast-changing landscape mosaic. To pursue this aim we built data-driven regression models of CO_2_ fluxes in the High-Arctic tundra of Svalbard. For this area, we showed that different, spatially heterogeneous drivers (mainly soil moisture and green fractional vegetation cover) acted to modify the dependences of GPP and ER on their classical drivers, namely solar irradiance and environmental temperature, thus determining the flux variability at site scale. Further studies should aim to create a solid framework of plant classifications according to their relationship with CO_2_ fluxes. A plant classification based on carbon fluxes would in particular benefit studies based on the Eddy Covariance method^42^. Maps of vegetation cover have been shown to be a crucial point in relating Eddy Covariance and chamber measurements, allowing for the explanation of measurement variations in relation to the characteristics of the footprint area, and for upscaling the results of chamber measurements to the footprint-scale^72,73^. This will help in small-scale assessment of carbon fluxes and associated climatic feedbacks.

## Methods

### Study area

The experimental site is located in the foremost part of the Bayelva river catchment, West of Ny Ålesund, in the Brøgger peninsula, Spitsbergen, Norway (78° 55′ 24″ N, 11° 55′ 15″ E), and can be classified as moderate snow-bed habitat^74^. The catchment, covering 32 km^2^ and ranging from 4 to 742 m a.s.l.^75^, is surrounded on the southern border by steep mountains that host the Austre and Vestre Brøggerbreen glaciers. The measurement site (Fig. 1) is located on a slight hill slope, degrading towards South to a small lake, on the Signehamna formation^76^. In 2016–2019 the ground was snow-free from June to September, the maximum snow depth occurred in April (27 cm) and July–August daily air temperature and relative humidity were 5.7 °C and 78.3% (see https://seklima.met.no/observations/). The mean annual (water equivalent) precipitation is 588 mm/year. The vegetation cover is heterogeneous, typical of the bioclimate subzone B-C^77^ with vascular plants constellating the matrix of mosses and lichens, and can be classified as prostrate dwarf-shrub and herbs tundra (P1)^65^. The soils are characterized primarily as haplorthels, with high lithic and low nutrient content^78^ and the underlying permafrost has typical active layer depths of ∼ 0.5 to 1.5 m^79^.

### Scopes and sampling design

We designed three different sampling strategies, aimed at investigating: (1) the temporal variability of CO_2_ fluxes, by performing 24-h measurements at intervals of about 2 h at a fixed sampling point, mostly covered with *Carex* (*point-scale samplings*); (2) the drivers of CO_2_ variability at the site scale (*site-scale samplings*, 177 measurements from 07/23/2019 to 08/03/2019), where the sampling points were randomly distributed over the sampling site, covering an area of about 22,000 m^2^ (Fig. 1); (3) whether and how different vegetation types affect fluxes (*species-specific samplings*), by performing measurements in points covered with the 5 most representative vascular species or genus in the catchment: *Carex* spp. (CX), *Dryas octopetala* (DR), *Salix polaris* (SL), *Saxifraga oppositifolia* (SX) and *Silene acaulis* (SI). Each vascular class was made of 15 measurements obtained by merging 8 measurements performed for this purpose and 7 points of the site-scale samplings (which was conducted in the same area) covered with the target species. When more than 7 points were available, a random subset of those was retained. Samples belonging to *point-scale sampling* were not included in *site-scale* and *species-specific samplings.* See Fig. S3 in supplementaries for measurement distribution along the campaign.

### Flux measurements

In all sampling sets we simultaneously measured CO_2_ fluxes and basic meteoclimatic and environmental variables. Fluxes were measured by the non-steady state, closed dynamic flux chamber method^80^ using a LI-COR LI-840 IRGA (InfraRed Gas Analyser) spectrophotometer and a circular stainless-steel collar (661 cm^2^ area, well within the range used for this site^40,41,45^) inserted into the soil just prior to the measurement (to a depth of about 2 cm) where to place the transparent chamber (10 cm height). Gasses inside the chamber volume were mixed and homogenized through a coiled, pierced tube connected to the outlet of the gas analyzer and a pressure equalizer kept the pressure inside the chamber at equilibrium with the environment. The tendency of CO_2_ concentration change inside the chamber was linearly interpolated (*R*^2^ varied between 0.73 and 0.99) to obtain the flux value (see Fig. S5 in the Supplementary Material and also^56^). At each sampling point we consecutively measured the NEE with the transparent chamber, and ER by covering the chamber with a black cover, and purging all the apparatus between the two consecutive measurements. GPP was obtained by subtracting the two measurements (GPP = NEE-ER), assuming that the environmental conditions varied negligibly during the sampling time (about 3 min). Conventionally, GPP has negative values (GPP $\le$ 0), ER is positive (ER $\ge$ 0), while NEE can be either positive, negative or null, depending on the prevailing component (ER or GPP).

### Dataset

We associated to each sampling point a value of NEE, ER, GPP, two set of meteoclimatic variables, averaged over the flux sampling time of NEE or ER respectively, an estimate of the Green Fractional Cover (GFC), the land cover classification, the time of sampling (h = hour + minute/60, that varied between 0.0 and 23.9; where ‘hour’ and ‘minute’ were the mean values over flux acquisition time) and the day of sampling (expressed as Day Of the Year, DOY, 0–365). During each flux acquisition, we also measured basic meteoclimatic variables. Air relative humidity (RH, %), air temperature (T_a_, ℃), and solar irradiance (rs, W/m^2^) were measured with a LSI Lastem class A pyranometer and thermo-hygrometer. Soil temperature (T_s_, ℃) and soil moisture (VWC, %) were measured at the base of the collar with PT-100 and SM150T sensors. Air pressure (Pr, hPa) was measured by a sensor installed above the flux chamber. The raw dataset was quality controlled by statistical analysis of the frequency distribution and by the Rosner tests^81^ to identify potential outliers. At each sampling point, we also collected 10 Mpixel RGB pictures at nadir, in order to monitor the composition and fractional cover of vegetation inscribed within the collar (see Fig. S1 in the Supplementary Material), by using a HUAWEI P20 PRO CLT-L29, equipped with Leica dual camera and CMOS sensor placed at an height of about 50 cm above the soil surface. The GFC was estimated from the pictures as the ratio of the vertical projection of the green vegetated area to the ground (total) area of measurement, by an ad-hoc algorithm using MatlabR2020a. Pictures were low-pass filtered by using ‘wiener2’ Matlab intrinsic function to reduce the signal-to-noise ratio and RGB channels allowed to obtain a greenness index (*g* = *2G-B-R,* with R, G and B the brightness of red, green and blue channel at each pixel) to discriminate green, photosynthetic vegetation form background (following^82^) inside the collar. Such an estimate does not account for vegetation layering, which is negligible in the Arctic tundra, and therefore GFC ranges between 0 and 1. Here, the area of the single measurements, corresponding to the round area included within the steel collar, will be called sampling point. Sampling site will refer to the area over which 177 measurements were performed in points having random spatial distribution. Points were georeferenced and pseudoreplicates having the same coordinates within the GPS uncertainty were discarded from the dataset of *site-scale* and *species-specific samplings*. Finally, in *site-scale samplings,* we classified the soil cover into 4 macro-classes: vascular species (V), non-vascular species (NV, including lichens, mosses and bacterial soil crust), bare soil (BS) and mix of species (MIX), the latest where it was not possible to define a clear predominance of either V or NV over the sampling surface. Vascular species were then divided into 5 classes: *Carex *spp. (CX), *Dryas octopetala* (DR), *Salix polaris* (SL)*, Saxifraga oppostifolia* (SX) and *Silene acaulis* (SI), subsequently selected for the *species-specific samplings* (https://svalbardflora.no/). Here, by vegetation cover we refer to the type of vegetation included within the sampled area (e.g. V, NV, BS, MIX), while by fractional vegetation cover or green fractional vegetation cover we refer to the GFC estimate, as explained above. The methods applied for vegetation classification and GFC estimation do not require the harvesting or manipulation of vegetation and are in compliance with relevant institutional, national, and international guidelines and legislation.

### Regression models

CO_2_ drivers were selected using multi regression models for the site samplings set and for the species-specific set, where Eqs. (1) and (2) reproduced only a small part of fluxes variability. We started from the known drivers of carbon emissions and uptake: air temperature, *T*_*a*_^29^, and solar irradiance, *rs*^30^, respectively, as in Eqs. (1) and (2). We assumed that the rs/PAR (Photosynthetically Active Radiation) ratio is constant^30^ and that wavelengths outside the PAR band are photosynthetically inactive^83^. Here, we chose the same model class selected by Magnani et al.^56^ for the Alpine tundra, based on the hypothesis that additional variables in the model cause small modifications of the parameters of Eqs. (1) and (2). Hence, using Taylor expansions, a general formulation of the multi-regression models can be written as

M1 $$ER = \left( {a_{0} + a_{1} x_{1} + a_{2} x_{2} + \cdots } \right)\exp\left(b_{0} T_{a}\right) + \varepsilon ,$$

M2 $$\begin{aligned} GPP & = [F\alpha_{0} \, rs/\left( {F + \alpha_{0} \, rs} \right)\left] \right[\left( {A_{0} + A_{1} y_{1} + A_{2} y_{2} + \cdots } \right) \\ & \quad + (F\alpha_{0}\, rs/\left( {F + \alpha_{0} \, rs} \right))\left( {B_{0} + B_{1} y_{1} + B_{2} y_{2} + B_{3} y_{1} rs + B_{4} y_{2} rs + \cdots } \right)] + \varepsilon \\ \end{aligned}$$

The additional variables, represented by *x*_*i*_ and *y*_*i*_ in (M1) and (M2), were selected among the measured ones, plus the hour of sampling (h), the sampling date (DOY) and the green vegetation cover (GFC), as described in ‘Dataset’. A partial-correlation analysis on all the candidate drivers was performed before building the model and candidate drivers having significant correlations were not allowed to be simultaneously included in the model. Different letters for the additional variables in Eqs. (M1) and (M2) have been used to highlight that the two sets of predictors were selected independently of each other. We used the set of meteoclimatic variables recorded during NEE measurements, as humidity and temperature do not significantly vary during the consecutive NEE and ER measurements, while it is important to use the *rs* records obtained simultaneously with NEE measurements, when photosynthesis is active. Parameters that gave non-significant (*p* > 0.05, see also ‘Statistical analysis’) contributions to the model were statistically pruned. In multi regression modelling, the use of an increasing number of variables could enhance model representativeness to the detriment of model simplicity. We quantified model representativeness by the explained variance, defined as ${{\sigma }_{expl}}^{2}= ({{\sigma }_{flux}}^{2}-{{\sigma }_{res}}^{2})/{{\sigma }_{flux}}^{2}$, with ${{\sigma }_{flux}}^{2}$ the variance of measured fluxes (either ER of GPP) and ${{\sigma }_{res}}^{2}$ the model residual variance (i.e. the variance of residuals, $\varepsilon$). The most efficient model was selected by means of the Akaike Information Criterion (AIC, ^33^), which identifies the model that minimizes the residual variance, with a penalty for the number of parameters. For regressive models, AIC is expressed as $AIC=N\mathrm{log}\left({\sum }_{i}{\varepsilon }_{i}^{2}\right)-2k$^34^, with *k* the number of estimated parameters, including the residual variance itself, and *N* the number of data. The smallest value of AIC identifies the most efficient model. Finally, we used the Lilliefors’ test^35^ for assessing the null hypothesis of gaussian distribution of the residuals, and Bartlett's test^36^ for assessing their heteroscedasticity. Once the model was identified, the weight of each additional drivers included in the regression was estimated by including one additional driver at a time in the regression and refitting the model, thus obtaining the explained variance for the reduced model, to be compared with the explained variance of the classical model (1) or (2). We performed the statistical analysis in MatlabR2020a, using intrinsic functions for nonlinear regressions, Lilliefors' and Bartlett's test.

### Statistical analysis

In the *species-specific sampling,* a preliminary analysis of the variables averaged over each vegetation species highlighted whether different species were associated with characteristic environmental and flux values. Significant differences (i.e. not ascribed to random fluctuations, *p* < 0.05) between species were assessed using a randomization technique that follows Jacobson et al.^84^: two series of species-specific data were shuffled, by mixing the individual values across the series, several times, then the difference of the means of the shuffled series (which is theoretically null) gave an estimate of the probability (P) of obtaining a difference larger than the unshuffled one. This allowed us to test the distribution of the difference of the means against the null hypothesis of statistically different means, by performing a double-tailed test. Randomization was also used to assess (i) the significance of regression coefficients, by shuffling pairs of dependent-independent variables and testing the null hypothesis of statistically uncorrelated variables, and (ii) the significance of the differences observed between species-specific models, by shuffling the independent variables for two series of measurements (e.g. dependent-independent pairs measured for two species), using the shuffled series to fit the models and testing the hypothesis that the observed difference was induced by random fluctuations of the variables. In the last case, the comparison of the parameters estimated for different vegetation classes was used to assess whether the species-specific response of the system to the same drivers was comparable (e.g. different species may have different specific productivity for the same GFC resulting in different CO_2_ uptake). We implemented ad hoc functions in MatlabR2020a for estimating P-values with the shuffling techniques. A detailed description of the statistical tests can also be found in Magnani et al.^56^.

## Supplementary Information

Supplementary Information.

## Data Availability

The dataset generated and analysed during the current study is available in the Zenodo repository (10.5281/zenodo.5815579).
