# Supplementary material for: Microscale drivers of summer CO2 fluxes in the Svalbard High Arctic tundra
Source: Sci Rep. 2022 Jan 14;12:763. doi: 10.1038/s41598-021-04728-0 (PMC8760244; doi:10.1038/s41598-021-04728-0)
Supplement: Supplementary file 1 — Supplementary Information. [file 41598_2021_4728_MOESM1_ESM.pdf]

Title: Microscale drivers of summer CO_2 fluxes in the Svalbard High Arctic tundra

Authors: Marta Magnani*[1,2], Ilaria Baneschi[3], Mariasilvia Giamberini[3], Brunella Raco[3], Antonello Provenzale[3]

Affiliations:
[1] Institute of Geosciences and Earth Resources, Via Valperga Caluso 35, 10125 Torino, Italy
[2] University of Turin & INFN, Via Pietro Giuria 1, 10125 Torino, Italy
[3] Institute of Geosciences and Earth Resources, Via Moruzzi 1, 56124 Pisa, Italy

Corresponding Author: Marta Magnani, marta.magnani@edu.unito.it , Via Pietro Giuria 1, 10125 Torino, Italy

Supplementary Material

Extended site description

The experimental site is located in the foremost part of the Bayelva river catchment, located in the Brøggerhalvøya peninsula, to the West of the research base of Ny Ålesund, Spitsbergen, Svalbard Archipelago, Norway (78055'24'' N, 11055'15''E), see Fig. 1. The catchment (about 32 km^2 ranging from 4 to 742 m a.s.l.) faces the Kongsfjorden at the north-eastern side, and it is surrounded by steep mountains at the southern border (from South-East to North-West), where the Austre and Vestre Brøggerbreen glaciers are located. The southern and eastern parts of the watershed are underlain by sedimentary and metamorphic rocks such as red sandstones, quartzite and phyllite, while the northern and western parts are characterised by sedimentary rocks such as sandstone, shale, dolomite and limestone[S1,S2]. The Bayelva river flows on a bedrock of moraine and terminates in a sandur consisting of boulders and gravel[S3]. The Bayelva catchment is underlain by permafrost, with active layer depths of ~0.5 to 1.5 m[S4,S5] and soils characterized primarily as haplorthels, with high lithic and low nutrient content[S6]. The catchment has been the object of numerous studies and detailed descriptions can be found for instance in Repp[S7], Hodson et al.[S8,S9], Killingtveit[S10], Lloyd[S11] and Nowak and Hodson[S4].

Referring to the three years prior to the samplings, the ground was mainly snow-free from June to September, corresponding to the summer growing season. The mean annual (summer, i.e. June to September) air temperature and relative humidity measured at the Ny Ålesund weather station (located 8 m a.s.l. and managed by the Norwegian Meteorological Institute) were respectively -2.4°C (5.7°C) and 70.9% (78.3%). Data are accessible via https://seklima.met.no/observations/. The maximum snow depth usually occurred in April, with peak values of 27 cm over the whole period, and the mean annual (water equivalent) precipitation was 588 mm/year[S12].

Within the watershed, the measurement site lies on a slight hill slope, degrading towards South to a small lake, on the Signehamna formation[S13] that can be classified as moderate snow-bed habitat according to Elvebakk[S14]. Climatic subzones B and C. The local vegetation can be classified as dry tundra with patchy vegetation (P1-CAVM Team 2003[S15]) characterized by prostrate shrubs (< 5 cm tall), such as Dryas and Salix spp., with graminoids and forbs. Lichens are also common[S16], sometimes also forming lichen and moss crust, as described by Mugnai et al.[S17]. Successional patterns were observed in the glacier foreland, with vegetation cover and soil carbon pools increasing with the progress of succession[S18,S19]. Vascular species cover also increases close to the fjord shores[S18,S19], where the measurement site is located.

References

*S*1. Orvin, A. K. Geology of the Kings Bay region, Spitsbergen: with special reference to the coal deposits. (1934).
S2. Svendsen, H. *et al.* The physical environment of Kongsfjorden–Krossfjorden, an Arctic fjord system in Svalbard. *Polar Res.* **21**, 133–166 (2002).
S3. Bogen, J. & Bønsnes, T. E. Erosion and sediment transport in High Arctic rivers, Svalbard. *Polar Res.* **22**, 175–189 (2003).
S4. Nowak, A. & Hodson, A. Hydrological response of a High-Arctic catchment to changing climate over the past 35 years: a case study of Bayelva watershed, Svalbard. *Polar Res.* **32**, 19691 (2013).

S5. Boike, J. *et al.* A 20-year record (1998–2017) of permafrost, active layer and meteorological conditions at a high Arctic permafrost research site (Bayelva, Spitsbergen). *Earth Syst. Sci. Data* **10**, 355–390 (2018).
S6. Wojcik, R., Palmtag, J., Hugelius, G., Weiss, N. & Kuhry, P. Land cover and landform-based upscaling of soil organic carbon stocks on the Brøgger Peninsula, Svalbard. *Arct. Antarct. Alp. Res.* **51**, 40–57 (2019).
S7. Repp, K. The Hydrology of Bayelva, Spitsbergen: Paper presented at the 7th Northern Res. Basins Symposium/Workshop (Ilulissat, Greenland May/June - 1988). *Hydrol. Res.* **19**, 259–268 (1988).
S8. Hodson, A., Tranter, M., Gurnell, A., Clark, M. & Hagen, J. O. The hydrochemistry of Bayelva, a high Arctic proglacial stream in Svalbard. *J. Hydrol.* **257**, 91–114 (2002).
S9. Hodson, A. J., Mumford, P. N., Kohler, J. & Wynn, P. M. The High Arctic glacial ecosystem: new insights from nutrient budgets. *Biogeochemistry* **72**, 233–256 (2005).
S10. Killingtveit, Å. *Water balance studies in two catchments on Spitsbergen, Svalbard*. (2004).
S11. Lloyd, C. R. On the physical controls of the carbon dioxide balance at a high Arctic site in Svalbard. *Theor. Appl. Climatol.* **70**, 167–182 (2001).
S12. Dahl, M. B. *et al.* Warming, shading and a moth outbreak reduce tundra carbon sink strength dramatically by changing plant cover and soil microbial activity. *Sci. Rep.* **7**, 1–13 (2017).
S13. Dallmann, W. K., Piepjohn, K. & Ohta, Y. Geological map of Svalbard 1:100,000, sheet B4G Reinsdyrflya. (2005).
S14. Elvebakk, A. A survey of plant associations and alliances from Svalbard. *J. Veg. Sci.* **5**, 791–802 (1994).
S15. Team, C. M., Walker, D. A. & Trahan, N. G. *Circumpolar Arctic vegetation map*. (US Fish and Wildlife Service, 2003).
S16. Słupianek, A., Wojtuń, B. & Myśkow, E. Origin, activity and environmental acclimation of stem secondary tissues of the polar willow (Salix polaris) in high-Arctic Spitsbergen. *Polar Biol.* **42**, 759–770 (2019).
S17. Mugnai, G., Rossi, F., Mascalchi, C., Ventura, S. & De Philippis, R. High Arctic biocrusts: characterization of the exopolysaccharidic matrix. *Polar Biol.* **43**, 1805–1815 (2020).
S18. Nakatsubo, T. *et al.* Ecosystem development and carbon cycle on a glacier foreland in the High Arctic, Ny-Ålesund, Svalbard. *J. Plant Res.* **118**, 173–179 (2005).
S19. Yoshitake, S. *et al.* Vegetation development and carbon storage on a glacier foreland in the High Arctic, Ny-\AAlesund, Svalbard. *Polar Sci.* **5**, 391–397 (2011).

Supplementary figures and tables

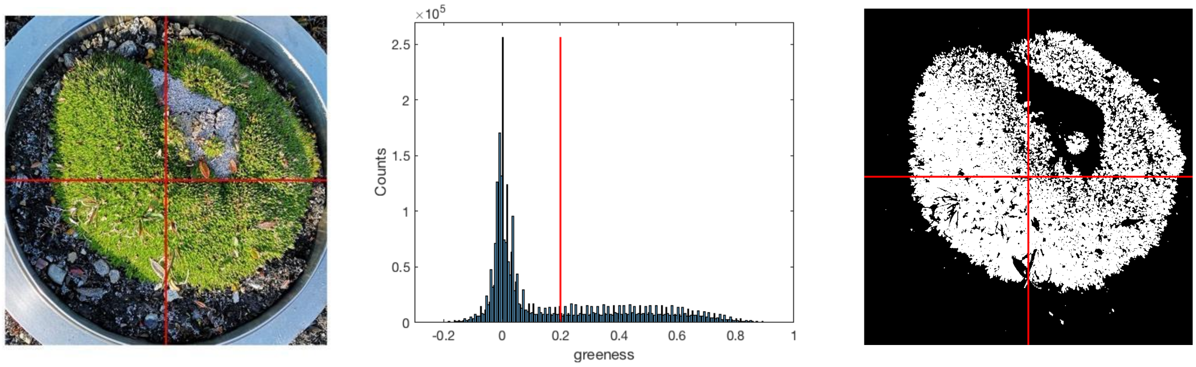

Figure S1. Example of Green Fractional Cover (GFC) estimation. Left: original RGB picture; Middle: greenness histogram with background threshold (red line); Right: greenness 'g' inside the sampling area (see Methods). Estimated GFC in this case was 0.63.

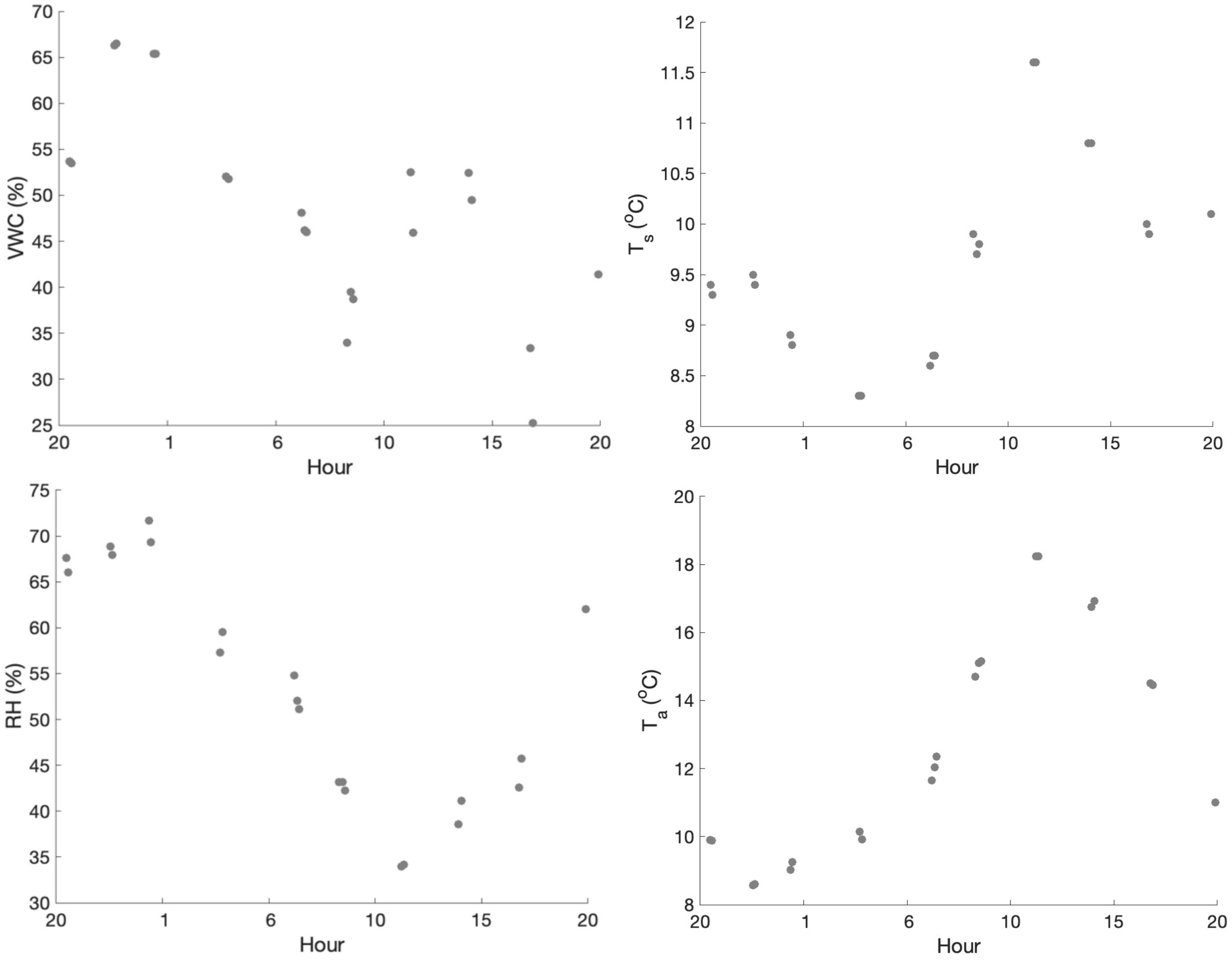

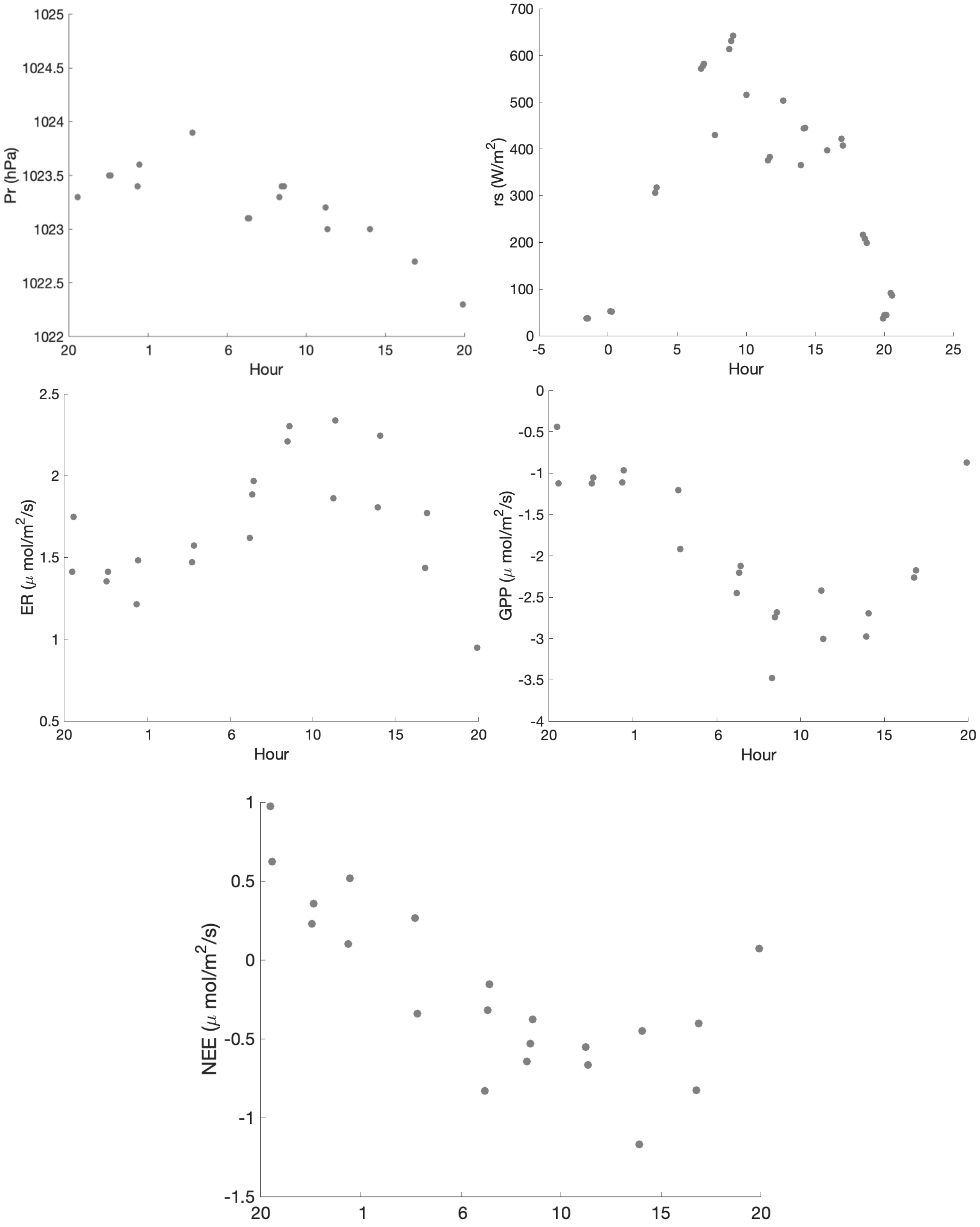

Figure S2. Temporal variation of soil moisture (VWC), soil temperature (T_s), air humidity (RH), air temperature (T_a), air pressure (Pr), solar irradiance (rs), Ecosystem Respiration (ER), Gross Primary Production (GPP) and Net Ecosystem Exchange (NEE) in the 24-h point sampling. Hours in UTC.

Veg. classes	DOY[a]	h[a]	Pr[b]	rs[c]	T_a[d]	T_s[d]	RH[e]	VWC[e]	GFC[f]	ER[g]	NEE[g]	GPP[g]
**CX-DR**	**-2.20 (0.03)**	-1.63 (0.17)	**7.41 (<0.01)**	66.59 (0.32)	**3.79 (<0.01)**	**1.56 (<0.01)**	**-17.27 (<0.01)**	**15.85 (<0.01)**	**-0.19 (<0.01)**	0.09 (0.89)	**1.20 (<0.01)**	**1.10 (0.04)**
**CX-SL**	**-2.47 (0.04)**	**-2.11 (<0.01)**	**9.73 (0.04)**	83.11 (0.12)	**2.38 (0.02)**	0.70 (0.22)	**-17.75 (<0.01)**	7.17 (0.07)	-0.04 (0.59)	**0.75 (<0.01)**	-0.18 (0.49)	**-0.93 (0.04)**
**CX-SX**	**-3.40 (0.01)**	-0.04 (0.84)	**8.35 (<0.01)**	68.10 (0.13)	**1.79 (0.04)**	0.51 (0.52)	**-11.81 (0.01)**	7.42 (0.06)	0.03 (0.81)	**0.92 (<0.01)**	-0.37 (0.08)	**-1.29 (<0.01)**
**CX-SI**	-0.47 (0.69)	**-2.37 (<0.01)**	**8.84 (<0.01)**	**74.57 (<0.01)**	**2.26 (<0.01)**	0.59 (0.55)	**-12.55 (<0.01)**	5.69 (0.05)	**-0.22 (<0.01)**	-0.29 (0.55)	**1.75 (<0.01)**	**2.04 (<0.01)**
**DR-SL**	-0.27 (0.67)	-0.48 (0.43)	2.31 (0.24)	16.52 (0.79)	-1.41 (0.17)	**-0.86 (0.02)**	-0.47 (0.89)	**-8.68 (0.01)**	**0.15 (<0.01)**	**0.66 (<0.01)**	**-1.38 (<0.01)**	**-2.04 (<0.01)**
**DR-SX**	-1.20 (0.59)	1.59 (0.15)	0.94 (0.64)	1.51 (0.98)	-1.99 (0.10)	**-1.05 (<0.01)**	7.75 (0.05)	**-8.43 (<0.01)**	**0.23 (<0.01)**	**0.82 (<0.01)**	**-1.57 (<0.01)**	**-2.40 (<0.01)**
**DR-SI**	**1.73 (0.04)**	-0.74 (0.39)	1.43 (0.16)	7.98 (0.79)	-1.52 (0.14)	**-0.97 (0.02)**	4.72 (0.59)	**-10.16 (0.02)**	0.03 (0.90)	0.08 (0.83)	-0.18 (0.76)	-0.26 (0.76)
**SL-SX**	-0.93 (0.29)	**2.06 (<0.01)**	-1.37 (0.51)	-15.01 (0.76)	-0.59 (0.53)	-0.19 (0.59)	8.22 (0.05)	0.25 (0.95)	0.07 (0.71)	0.17 (0.37)	-0.19 (0.47)	-0.36 (0.34)
**SL-SI**	**2.00 (0.04)**	0.26 (0.68)	-0.89 (0.61)	-8.54 (0.36)	-0.12 (0.21)	-0.11 (0.85)	5.19 (0.65)	-1.48 (0.61)	**-0.18 (0.01)**	**-1.04 (0.045)**	**1.93 (0.02)**	**2.97 (0.02)**
**SX-SI**	**2.93 (<0.01)**	**-2.32 (0.02)**	0.49 (0.72)	6.47 (0.16)	0.48 (0.61)	0.08 (0.81)	-3.03 (0.20)	-1.73 (0.62)	**-0.25 (<0.01)**	**-1.21 (0.01)**	**2.13 (<0.01)**	**3.33 (<0.01)**

[a] *Day of the Year (DOY, 1-365) and hour of measurement (h,0.0-24.9)*

[b] *Atmospheric pressure (Pr), in hPa*

[c] *Solar irradiance (rs) in W/m^2*

[d] *Air temperature (T_a) and soil temperature (T_s) in ℃*

[e] *Air humidity (RH) and soil humidity (VWC) in %*

[f] *Green Fractional Cover (GFC), between 0-1*

[g] *Ecosystem Respiration (ER), Gross Primary Production (GPP) and Net ecosystem Exchange (NEE), in $\mu mol/m^2/s$*

Table S1. Differences between variables measured at points covered with Carex spp. (CX), Dryas Octopetala (DR), Salix Polaris (SL), Saxifraga Oppostifolia (SX) and Silene Acaulis (SI). Difference between species 'i' and 'k', labelled as '$species_i$-$species_k$', corresponds to the difference between the average value recorded for species 'i' and the average value recorded for species 'k'. Probability P-value computed with the shuffling technique in bracket and significant value (P<0.05) highlighted in bold.

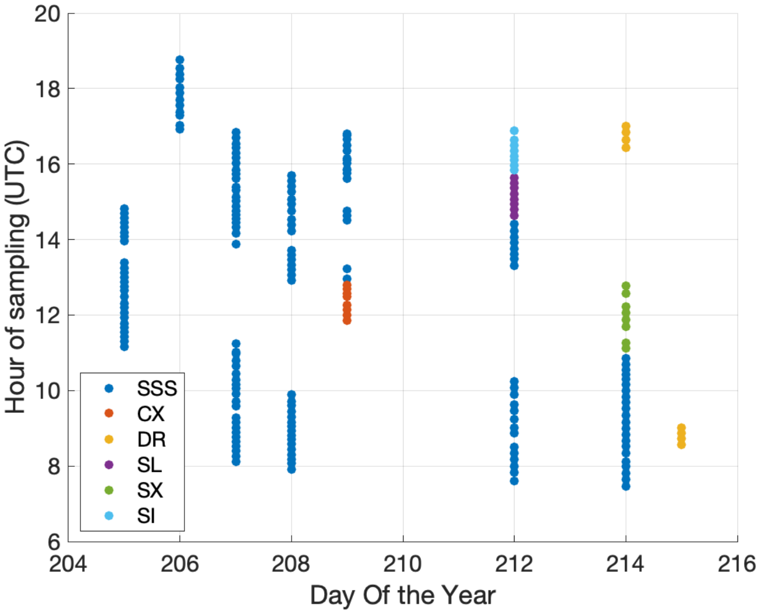

Figure S3. Distribution of site-scale samplings (SSS, blue) and species-specific samplings along the sampling campaigns. For species-specific samplings: CX=Carex spp., DR=Dryas Octopetala, SL=Salix Polaris, SX=Saxifraga Oppostifolia and SI=Silene Acaulis.

species	CX	DR	SL	SX	SI
CX		a_1	a_1, A_1	a_1, A_1	
DR	a_1		a_1, A_1	A_1	
SL	$a_1,\ A_1$	a_1, A_1			$a_1,\ A_1$
SX	a_1, A_1	A_1			$a_1\ , A_1$
SI			a_1, A_1	$a_1,\ A_1$	

Table S2. Parameters of Equations (3-4) having significant differences between species: Carex spp. (CX), Dryas octopetala (DR), Salix polaris (SL), Saxifraga oppositifolia (SX), Silene acaulis (SI)

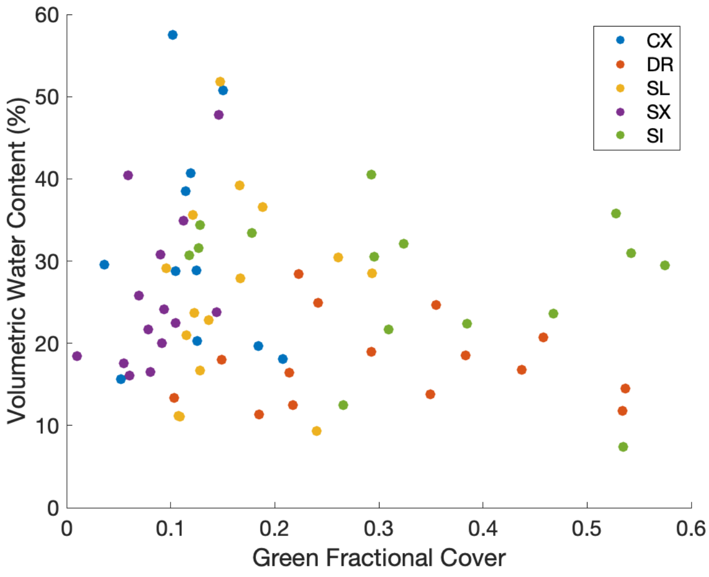

Figure S4. Soil Volumetric Water Content (VWC) versus Green Fractional Cover (GFC) for species-specific samplings: CX=Carex spp., DR=Dryas Octopetala, SL=Salix Polaris, SX=Saxifraga Oppostifolia and SI=Silene Acaulis.

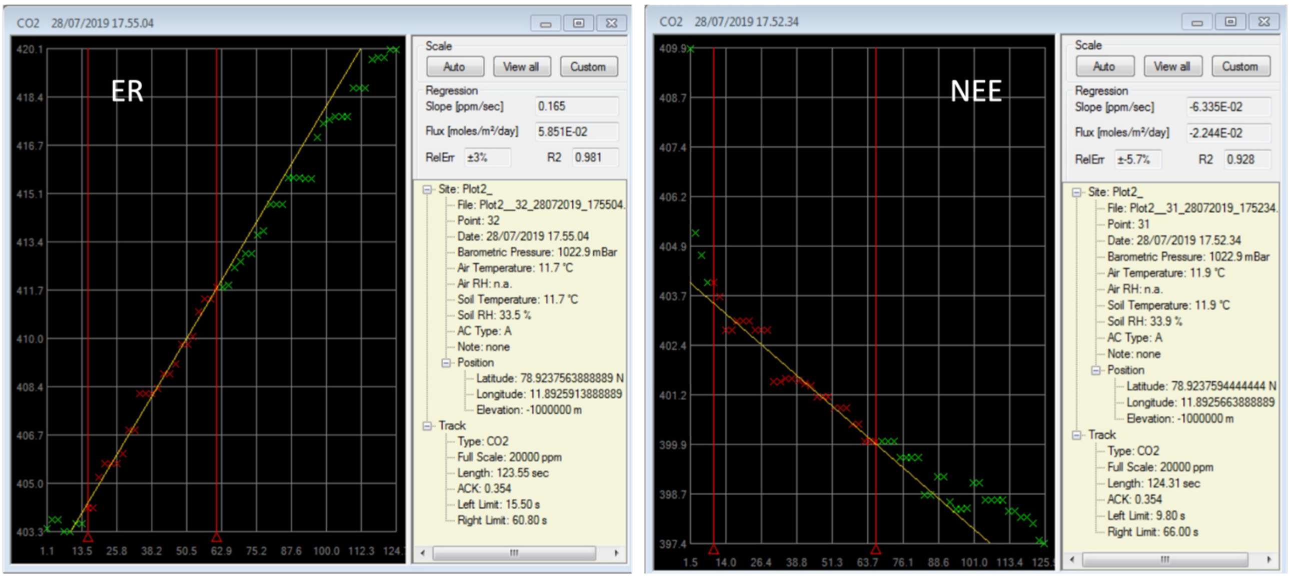

Figure S5. Example of flux measurement (as visualized by FluxRevision4.14 software) belonging to the site-scale sampling set and classified as mixed vegetation (MIX). Left: Ecosystem Respiration (ER) measured with the shaded chamber. Right: Net Ecosystem Exchange measured with the transparent chamber.
